# Ontology-Driven Monitoring of Patient's Vital Signs Enabling Personalized Medical Detection and Alert

**DOI:** 10.3390/s140101598

**Published:** 2014-01-17

**Authors:** Anna Hristoskova, Vangelis Sakkalis, Giorgos Zacharioudakis, Manolis Tsiknakis, Filip De Turck

**Affiliations:** 1 Internet Based Communication Networks and Services, Department of Information Technology—iMinds, Ghent University, Gaston Crommenlaan 8 Box 201, Ghent 9050, Belgium; E-Mail: filip.deturck@intec.ugent.be; 2 Institute of Computer Science, Foundation for Research and Technology and the Department of Computer Science, University of Crete, Vassilika Vouton, P.O. Box 1385, Heraklion 71110, Greece; E-Mails: sakkalis@ics.forth.gr (V.S.); gzaxar@ics.forth.gr (G.Z.); 3 Department of Informatics Engineering, Technological Educational Institute of Crete, Estavromenos 71004, Greece; E-Mail: tsiknaki@ics.forth.gr; 4 Computational Medicine Laboratory, FORTH-ICS, N.Plastira 100, Vassilika Vouton, Heraklion 71110, Greece

**Keywords:** medical workflows, ambient intelligence (AmI), congestive heart failure (CHF), semantic reasoning, medical alarms, quality of service (QoS)

## Abstract

A major challenge related to caring for patients with chronic conditions is the early detection of exacerbations of the disease. Medical personnel should be contacted immediately in order to intervene in time before an acute state is reached, ensuring patient safety. This paper proposes an approach to an ambient intelligence (AmI) framework supporting real-time remote monitoring of patients diagnosed with congestive heart failure (CHF). Its novelty is the integration of: (i) personalized monitoring of the patients health status and risk stage; (ii) intelligent alerting of the dedicated physician through the construction of medical workflows on-the-fly; and (iii) dynamic adaptation of the vital signs’ monitoring environment on any available device or smart phone located in close proximity to the physician depending on new medical measurements, additional disease specifications or the failure of the infrastructure. The intelligence lies in the adoption of semantics providing for a personalized and automated emergency alerting that smoothly interacts with the physician, regardless of his location, ensuring timely intervention during an emergency. It is evaluated on a medical emergency scenario, where in the case of exceeded patient thresholds, medical personnel are localized and contacted, presenting *ad hoc* information on the patient's condition on the most suited device within the physician's reach.

## 1. Introduction

During the past decade, technology has gradually been moving to the concept of ambient intelligence (AmI), in which smart environments help inhabitants in everyday life. AmI supports the pervasive diffusion of intelligence in the surrounding environment, through various wireless technologies (Zigbee, Bluetooth, RFID, WiFi) and intelligent sensors. The first applications appearing in the clinical domain were mainly focused on addressing the need to better support remote patient monitoring (vital sign monitoring [1], soft copy radiological film review [2]) and to provide condition-specific diagnostics and treatment [3]. Such e-health applications and wireless medical devices can significantly improve the quality of healthcare and promote evidence-based medicine.

Later, the need became apparent to provide services able to interconnect all the fragmented available e-health and automation systems, in order to realize integrated platforms facilitating time-critical care in case of an emergency. Additionally, computerized AmI environments are extremely data-intensive, resulting in enormous databases. For example, it is generally assumed that every patient in an intensive care unit generates around 16.000 different values on a daily base. The amount of data and the heterogeneity calls for automated data processing. AmI environments should be able to facilitate the abstraction of the relevant information and support the physicians through smart medical decision support services. In addition, the optimal use and visualization of the medical data in gaining valuable insights into the patients condition has been an important research topic in recent years. It is expected that in future AmI environments, hundreds of medical support services will be active simultaneously in order to optimize the care of critically ill patients. With an increased deployment of these services, reusing existing services as building blocks to create new ones not only offers more flexibility to the developer, but accelerates also the design process, because the existing parts are already extensively validated. In this direction, the AmI framework presented in this paper is designed based on the principles of service-oriented architectures (SOAs), wherein all components are implemented as web services. SOAs support a generic communication system in which services can easily be plugged in. AmI provides efficient management and data subscription for medical support services. Medical measurements from laboratories or monitors are processed by the AmI framework, and depending on the priority, the results are sent to the physicians smart phone. The novelty of the developed AmI framework proposed in this paper is: (i) the *personalized monitoring* of chronic (heart) disease patients able to detect the patient's health status and risk stage based on the results from the well-established Framingham Heart Study [4]; (ii) the *intelligent alerting* of the dedicated physician in case of an emergency through the automatic composition of medical service workflows, gathering all the required data; (iii) the *dynamic adaptation* of the full vital sign monitoring environment on any available device located in close proximity to the physician or even his/her smart phone. The intelligence of such a service lies in the ontology-based modeling of the patient's and physician's context and the available medical devices, which will be further elaborated in Section 5. Additionally, the AmI framework implements three stages in supporting the use of complex medical workflows. These include: (i) the translation of services into rules enabling a rule-based system, which triggers events based on new data; (ii) the construction of a service workflow in order to gather all the necessary information in notifying the physician of the patients state; and (iii) the mapping of the service workflow to the available devices for execution depending on the location of the physician and their quality attributes, such as location, screen size and on-status. In order to achieve such functionality, the following devices and technologies are available in our paradigm, detailed in Section 6.1:

- *Wireless medical devices and sensors*acquiring patient's vital signs.
- An *Indoor Localization System*(ILS) consisting of a network of sensors used as anchor points in order to pinpoint the location of a person.
- A *Monitoring application* [5] recording the aforementioned patient bio signals and hosting risk assessment algorithms to enable the alerting process.
- An *ontology-driven*application intelligence capable of reasoning on the patient's congestive heart failure (CHF) profile and the available devices for monitoring and notification.

The remainder of the paper is structured as follows: Section 2 provides an overview of existing frameworks supporting the real-time monitoring of patients. In order to illustrate the AmI framework, Section 3 introduces a general clinical scenario related to monitoring patients diagnosed with congestive heart failure (CHF). Subsequently, Section 4 elaborates on the implementation details of the architectural components of the AmI followed by a description of the developed ontologies for the formal definition of the medical patient profiles and the specification of device characteristics in Section 5. Section 6 describes the validation of the clinical scenario, including the supportive technologies specific to our implementation. A discussion on the benefits of a semantically-enhanced remote monitoring AmI is presented in Section 7. Finally, Section 8 ends with concluding remarks and future improvements.

## 2. Related Work

Advanced decision support systems are in use at a handful of academic medical centers [6–8]. Their predefined rules generate reminders to physicians based on clinical data, such as laboratory results, visit diagnosis, coded medications prescribed in the clinic and vital signs collected on encounter forms. Software alerts track patients’ vital trends and intervene on time before complications occur. Such facilities linked via telemedicine and computer monitors to the hospital rooms provide for the required around-the-clock specialized care of hundreds of patients. Although this approach emphasizes direct physician interaction and extensive clinical decision support, it lacks support for complex scenarios capturing multiple chronic conditions of a patient.

The Intensive Care Information System of the ICUin Ghent [9,10] facilitates the abstraction of relevant information and supports the physicians through several medical support services, such as kidney dysfunction based on the RIFLE criteria (Risk Injury Failure Loss End-Stage Kidney Disease), CPIS (Clinical Pulmonary Infection Score), prescribing antibiotics and calculating the SOFA score (Sequential Organ Failure Assessment score) as predictors for the patients’ outcome. Laboratories and monitors provide medical data for processing, and depending on the priority, the results are sent to the patients’ bedside terminal, the physicians’ tablet or e-mail address. The UCHS (Ubiquitous Context-Aware Healthcare Service System) [11] context-aware decision support system uses RFID sensors to sense users life vital signal, such as electrocardiogram (ECG/EKG), heart rate (HR), respiratory rate (RR), blood pressure (BP), blood sugar (BS), temperature and light. Medical expert suggestions are translated into a medical ontology, which provides users requirements inference and a relative services search in a UDDIrepository by a semantic inference engine. Similarly, CAMPH (Context-Aware Middleware for Pervasive Home Care) [12] offers several key-enabling system services that consist of P2P-based context query processing, context reasoning for activity recognition and context-aware service management. It can be used to support the development and deployment of various home care services for the elderly, such as patient monitoring, location-based emergency response, anomalous daily activity detection, pervasive access to medical data and social networking. CAMPH is aimed to support autonomous physical spaces owned by different organizations, which enables the elderly to interact or be in touch with care-giving stakeholders “living” in different geographical spaces. The work in [13] presents the design of a mobile gateway for independent life and e-health support. The gateway is deployed on the person's smartphone, which reads measurements and sends alarms based on a rule-based system in order to detect anomalies. It uses OSGiin order to deploy several services, such as bio-medical parameter monitorization, alerts and communication with a coordination center.

Commercial clinical decision support products are also supported by Philips Healthcare, such as Neonatal Event Review, ProtocolWatch, On-line Electronic Help (OLEH), Philips IntelliVue and Event Surveillance. Neonatal Event Review [14] detects documents and provides management information on apnea, bradycardia, and desaturation as described by physicians. The recorded information is provided through the bedside recorder or (central) printer. ProtocolWatch [15] continually monitors a single value, e.g., sepsis, against care protocol criteria from the bedside monitors. Whenever criteria are met, the program prompts clinicians for the tests, observations or interventions indicated by the protocol and produces a log that can be printed for documentation and quality improvement. OLEH [16] provides real-time access to critical anesthesia-related information on the patient monitoring screen. All these applications are accessible on the Philips IntelliVue patient monitors [17]. These monitors provide caregivers on the general floor with direct access to an automated scoring system based on the hospital's policies. This enables the manual and automatic creation of events during predefined clinical situations through an Event Surveillance application [18] that identifies and documents clinically significant patient episodes for review.

Apart from commercial systems, numerous EU projects focus on monitoring the vital signs of (elderly) patients, mostly at home [19–23]: OLDES (Older People's e-Services at Home), CAALYX (Complete Ambient Assisting Living Experiment), K4CARE (Knowledge-based Home Care eServices for an Aging Europe), ENABLE(a wearable system supporting services to enable elderly people to live well, independently and at ease), SOPRANO (Service-Oriented Programmable Smart Environments for Older Europeans), REACTION (Remote Accessibility to Diabetes Management and Therapy in Operational Healthcare Networks), INHOME(an intelligent interactive services environment for assisted living at home), MonAMI (Mainstreaming on Ambient Intelligence) and Hydra (networked embedded system middleware for heterogeneous physical devices in a distributed architecture). These platforms transmit data on the health status, risk assessments and self-management incitements from sensors and devices at the patient's home to health-care professionals, informal carers and emergency and crisis management teams. Additionally, PERSONA (Perceptive Spaces Promoting Independent Aging) [24] and UniversAAL (ambient assisted living (AAL)) [25] provide solutions for user activity monitoring. PERSONA utilizes sensor data fusion and the semantic specification of user actions. It applies reasoning based on probabilistic rules for human activity detection by combining several activity descriptions into a human behavior description. UniversAAL aims at an open platform that provides a standardized approach, making it technically feasible and economically viable to develop ambient assisted living (AAL) solutions. It defines hardware and software infrastructure for smart environments, called AAL spaces, which enable context sharing and reasoning about activities carried out by the assisted person. The runtime environment is responsible for the deployment of the purchased services from a uStore and the communication between the different devices.

Two legacy research projects of the German Federal Ministry of Education and Research, EMBASSI [26,27] and its follow-up, DynAMITE [28], focus on infotainment systems at home, in automobiles and in terminal systems. The projects support the multimodal interaction between devices and software components of different software producers in order to analyze the interaction of users, to interpret the users’ goals and to realize those goals by using the interoperability abilities of the whole device ensemble. For this purpose, a context model is used to define the system view of the world and PDDLas the meta-language to define device functions. Both projects do not use semantics, merely PDDL as a meta-language, and the planning domain does not consider device quality of service (QoS) parameters.

Although these platforms fulfill the desired remote monitoring functionality, they generally lack support for: (i) personalized automatic analysis of the medical profile of the patient; and (ii) flexible combination of the available medical services and devices into complex medical workflows. The current article presents the main engineering underpinning of an AmI framework characterized by significantly enhanced flexibility and extensibility compared to the current state-of-the-art, thus offering physicians the possibility for remote event detection of a critical patient state through the support of semantically defined disease profiles and custom rules. Semantic patient profiles enable the automatic classification of patients into specific disease (CHF) risk stages, depending on their history and current state, personalizing the provided healthcare. The implemented planning algorithms automatically assemble medical workflows out of existing semantically enriched (medical) services, providing the responsible physician with timely and complete information on the patient's state. Dynamic notification mechanisms take into account the physician's location and succeed in remotely displaying the full vital sign monitoring application on the device of his/her choice, in order to ensure, in time, intervention in case of an emergency. The proposed framework responds to a changing context, such as physician location, new services/devices, new disease specification rules and the failure/overload of the network infrastructure, by adapting the medical workflows at runtime.

## 3. Vital Signs Monitoring and Alert Detection Scenario

In order to validate our dedicated AmI framework, a patient monitoring and physician notification scenario is implemented. Sections 4 and 5 detail the developed components supporting the required features, and Section 6.2 addresses the evaluation of the actual scenario.

**Figure 1.**
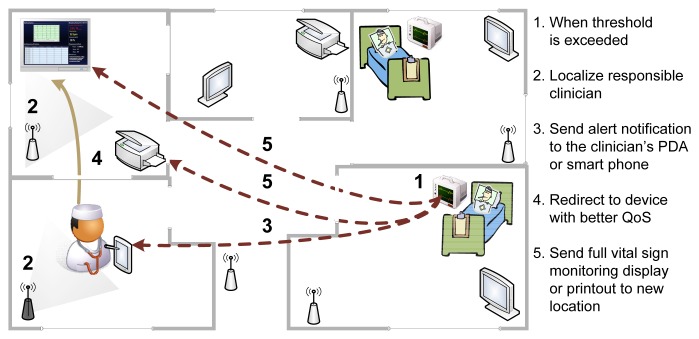
Localization and notification of the responsible physician during an emergency.

During everyday execution, the AmI monitoring environment receives and processes real-time patient data, detecting possible deviations from normal values. When a threshold is exceeded (Figure 1, Step 1) the responsible physician is localized (Step 2) and alerted (Step 3). Any surrounding devices, capable of displaying vital signs, are identified, and depending on their characteristics, a summary or complete overview of the patient's state is provided. Subsequently, the physician is redirected to a larger display (Step 4) in order to analyze, in detail, the vital signs (Step 5).

The alert detection parameters and *default thresholds* are illustrated in Table 1. In the case of exceeded thresholds, medical personnel are localized and contacted, presenting *ad hoc* information on the patient's condition on any device within the physician's reach. In order to further tailor the system to the patient's medical profile and assist physicians in selecting people who are predisposed to coronary disease, hypertension or valvular heart disease, we built, in Section 5.2, a personalized CHF-related risk profile. It is based on the risk appraisal function proposed in [29] that supports the Framingham Heart Study [4] (486 heart failure cases during 38 years of follow-up). The risk prediction estimates of the Framingham Heart Study for the risk of chronic heart disease are available as score sheets and direct risk functions. The choice of the appropriate risk prediction algorithm takes into account the following components: cardiovascular outcome, population of interest, time horizon and risk factors.

The utilized predictors are based on *age*, *coronary heart disease* and *valve disease status* provided by the patient electronic health record (EHR), as well as on *heart rate* (HR), *blood pressure* (BP) and *Body Mass Index*(BMI) provided by or calculated using the pulse oximeter, the BP monitor and the weight scale, respectively. The calculated risk probability is used to alter the *default threshold* values for each individual patient (a higher risk probability adds more constraints on the physiological patterns presented in Table 1), automatically classifying him/her in a specific CHF risk stage, resulting in personalized monitoring.

## 4. Implementation Details of the Ontology-Driven AmI Monitoring Framework

**Figure 2.**
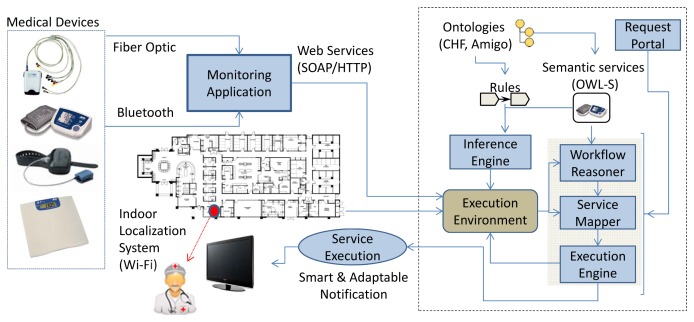
Ambient intelligence (AmI) patient monitoring and emergency detection framework architecture.

**Figure 3.**
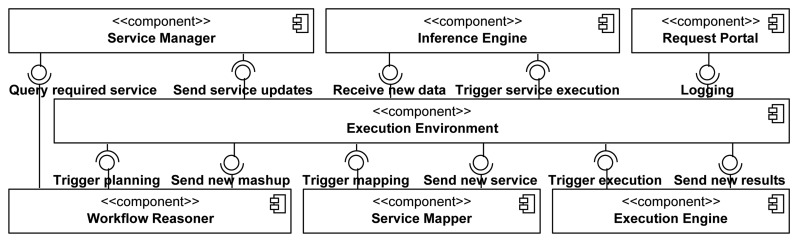
Workflow planning and service execution architecture.

The main building blocks of the AmI framework for monitoring patients with chronic heart conditions are presented in Figure 2. It is designed based on the principles of service-oriented architectures (SOAs) [30], wherein all medical components and devices are implemented as web services. The web service technology enables the reuse of services for gathering medical data values from patient monitoring devices. Depending on the defined or automatically adjusted thresholds and CHF risk stages, discussed in the previous section, the acquired patient measurements are sent to the physician's tablet, a nearby TV screen or even a printer for ECG printout [10] depending on his location. When needed, medical services are also invoked on request in order to query for overviews of historical results. This scenario is realized by the architectural components in Figure 3, which highlight the novel contribution of the AmI through semantic reasoning on the patient's profile (Section 4.1), automatic workflow composition (Section 4.2) and adaptable context-aware service execution (Section 4.3).

### 4.1. Personalized Automatic Classification of Patients in CHF Risk Stages

In order to enable the automatic classification of patients in CHF risk stages and the context-aware physician notification, the semantically defined *device* and *service ontologies* in Section 5.1 are managed by the **service manager**, while the *AmI ontology* from Section 5.2 is loaded in the **inference engine**.

The **service manager** is a repository of the available AmI services, including their OWL-S descriptions and the specific devices they run on. This enables the **Workflow Reasoner** to query for a specific service functionality during the workflow composition and the **service mapper** to select a corresponding device offering required QoS (availability, screen size, location) based on the user's context. The novelty of the presented AmI framework is the automatic translation of a service into a state-based SWRL (semantic web rule language) rule; IF *service-preconditions THEN service-effects*. These *service rules* and additional *user-defined rules* from the Framingham Heart Study classification defined in the *AmI ontology* are uploaded to the **inference engine**. The following example of a *user-defined rule* states that a *heart rate lower* than a default value of “40 bpm” is considered to be irregular.



Heart_Rate (?heartrate), Patient (Anna),hasMeasurement (Anna,?heartrate),lessThan (?heartrate,40),-> hasBradycardia (Anna,?heartrate)


If a *NotifyPhysician service* is available, it is automatically translated beforehand into a SWRL *service rule* by the **service manager** and added to the **inference engine**. This new *service rule* specifies that whenever a medical threshold is exceeded, a *NotifyPhysician service* should be executed (AtomicProcess (NotifyPhysician)), providing the responsible physician with the necessary patient information (hasDeviceContent (Notify- Physician,?measure)).



Medical_Measurement (?measure), Threshold (?threshold),hasThreshold (?measure, ?threshold),greaterThan (?measure, ?threshold)-> AtomicProcess (NotifyPhysician),hasDeviceContent (NotifyPhysician,?measure)


Similarly, whenever the automatic classification in a CHF risk stage is effective, a *NotifyPhysician service* having a HFClassification (?patient, Framingham_Heart_Study_classification) as precondition will be triggered for execution.



Patients (?patient),HFClassification (?patient,Framingham_16),hasDiagnosis (Framingham_16,Heart_failure)-> AtomicProcess (NotifyPhysician),hasDeviceContent (NotifyPhysician,Heart_failure)


These are two ways of registering a specific emergency: (i) through a *user-defined rule*, updating the patient risk profile with the specific medical condition; and (ii) a *service rule* triggering an actual action, such as the notification of his physician. The result is a data-driven AmI framework that reacts at runtime on new events flowing into the **monitoring application**.

### 4.2. Data-Driven Service Composition

The main contribution of the AmI framework is the forward chaining functionality of the **inference engine**, which uses Pellet reasoning on new patient measurements to evaluate the rules. Whenever a service precondition or a *user-defined rule* is satisfied by the available data, the **inference engine** triggers the execution of that service. For example, the *NotifyPhysician service rule* has as a result AtomicProcess (NotifyPhysician). This is an explicit definition of an OWL-S service using an *AtomicProcess* concept, which is used by the **inference engine** to define that the specific service should be executed, due to the met preconditions. The result is the registration of an event in the **execution environment**, triggering the execution of this service.

The **execution environment** manages the processing steps of the monitoring and alerting framework. It is designed as an event channel, enabling the rest of the AmI components to subscribe for specific events triggered by this module. Supported events are the addition/removal of (medical) services and devices, service/device failures, activation of rules triggering the execution of specific services (e.g., exceeded thresholds, CHF risk stages) and altered device QoS parameters (availability). In addition, it provides a feedback mechanism that executes services using incoming data in the **inference engine** and produces service results from the **execution engine** back to this environment. This results in a dynamic framework, where new knowledge is inferred at runtime, keeping track of the current user context.

Services scheduled for execution are passed on to the **workflow reasoner**, which decides if additional data is required for their execution. For example, the *NotifyPhysician service* expects patient profile information and contact details of the responsible physician in order to send a notification, such as a message or an e-mail. This is extracted from the execution of additional services, such as a *PatientRecord* and *UserLocation*. A medical workflow is automatically constructed through the forwarding of data between services using the HTN (hierarchical task network) planning described in [31]. The basic idea of this planning algorithm is the semantic matching of OWL-S service descriptions through backward chaining; a service result is used as the input required for the execution of another service. This realizes the composition of services into a workflow, gathering the necessary information on the specific patient risk profile, the contact details of his responsible physician and localizing an available device for displaying the data. It is a goal-driven approach, as opposed to the data-driven functionality of the **inference engine**.

Afterwards, the **service mapper** maps a corresponding *device* to each of the workflow service nodes, offering the required QoS able to execute the specific service, which is discussed in the following section.

### 4.3. Context- and QoS-Aware Device Selection and Service Execution

As mentioned before the **service manager** is a repository of the available AmI services and their OWL-S descriptions. It enables automatic querying for a required service functionality or device, offering specific QoS (availability, screen size, location), as defined in Section 5.1.2. As multiple devices offer semantically equivalent services (OWL-S descriptions), they are grouped into a single semantic service type, thus reducing the search space of available devices for service execution. For instance, both a TV, tablet and smart phone offer similar visualization services.

**Figure 4.**
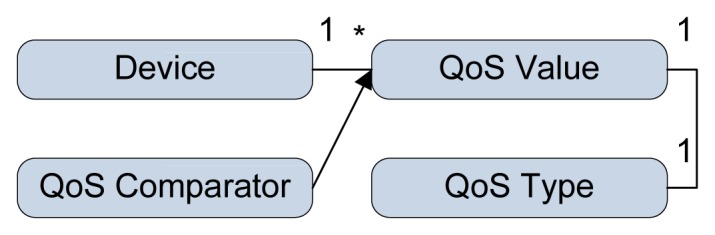
Definition of the quality of service linked to a specific device.

Comparison of the different devices able to execute the same service is based on the semantically defined QoS parameters describing the device properties. Examples include the average execution time, availability, screen size and location. They are defined beforehand, or their values are dynamically adjusted by the **execution engine** based on previous invocations. For example, the average execution time is updated after each invocation of a service on the device. The QoS of a specific device consists of a QoS type, such as the availability or execution time. Each QoS type has a QoS value and specific QoS comparator comparing the actual QoS values (Figure 4). These QoS types are assigned different priorities (‘on’ *status* has higher priority than *bigger screen size*) through the use of weights on the QoS Value. After the composition phase, the **service mapper** selects a specific device for each service of the constructed medical workflow based on the comparison of the weighted QoS values calculated by the QoS comparator. This offers an application manager with the possibility to extend the framework with new QoS parameters, define customary comparison techniques and specify different priorities of the QoS parameters favoring one or more of them.

The medical workflow constructed by the **workflow reasoner** is translated into an executable composite process by the **service mapper** through a specification of the data and controls bindings between the individual services. For each service node, an actual executable service instance is selected, depending on the device capabilities it is deployed on and the physician's context (e.g., location). For example, usually, a notification is sent to his tablet. Through device comparison of QoS properties, such as screen height and width, additional services are triggered to summarize the patient data for a small screen and supply redirection to devices with better screen quality. Whenever the physician changes his location by moving to a different room, a different device is selected to execute the *NotifyPhysician service* providing for a better screen size.

This dynamic execution is supported by the **execution engine** handling the invocation workflow of the services. It integrates an external library, the OWLS-API3.0 [32], for the service execution. The OWLS-API provides a Java API for programmatic access to create, read, write and execute OWL-S atomic and composite services. The API supports OWL-S version 1.2 by default. The service results are added to the **inference engine**, enabling a constant update of the current user context. Additionally, the **execution engine** supports recovery from failure. In the event of a service malfunction, an alternative device disposing of a semantically equivalent service is requested from the **service mapper**. If no equivalent services exist, the **workflow reasoner** partially reconstructs the failed part of the workflow.

In order to support transparency for a technical user, such as an administrator, a **request portal** visualizes the medical workflows, activated devices and service results.

## 5. Ontology-Driven Alert Detection

An AmI framework monitoring patients with chronic conditions requires: (i) *interoperability* between the various heterogeneous devices and technologies; and (ii) *personalized monitoring* of patients based on their medical profile and real-time measurements. The provided functionality and QoS parameters by the available devices and services should be automatically discovered based on semantically-defined features and executed depending on the current user context. Additionally, an intelligent behavior should emerge through the notion of this user (patient or physician) context, allowing for a smart and personalized combination of the available resources, providing for the necessary notification mechanisms. The patient context is defined as the patient's personal profile and medical record, including CHF risk stages and thresholds, which will result in a personalized diagnosis of his state. The responsible physician's context comprises of his profile, contact information, personal tablet and up-to-date location, enabling the in-time notification, presenting *ad hoc* information on the patient's condition on the most suited device within his reach.

### 5.1. Device and Service Ontology

As stated before, contacting a physician requires, from one point of view, the automatic combination of services, providing for the latest patient state, and from the other, the intelligent selection of the right devices for executing the actual notification. The proposed solution in this paper uses ontologies in order to formally define service functionality (Section 5.1.1) and device QoS parameters (Section 5.1.2).

#### 5.1.1. Semantic Description of Service Functionality

The semantic annotation of service functionality presents several advantages. It enables the reuse of such interfaces by several equivalent services. Additionally, services can be automatically discovered and queried based on this description. This results in the development of planning algorithms able to automatically discover, semantically match and compose services into complex workflows, accomplishing specific goals.

In the presented AmI framework, the available (medical) services are enriched with semantic annotations using OWL-S 1.2 [33]. Instead of defining their inputs and outputs using XMLschema data types, much like WSDL, they are expressed through ontological concepts in OWL. This introduces the notion of meaning to the available service functionality. In addition, in contrast to the WSDL description of inputs and outputs, OWL-S enables the specification of service preconditions required for the execution of a service and the effects of its actual execution. For the definition of these preconditions and effects, OWL-S supports the use of SWRL (semantic web rule language) expressions and built-ins (SWRLB), such as comparisons (equal, less than, greater than, *etc.*), math functions (add, subtract, multiply, divide, *etc.*) [34]. SWRL expressions are used for coding procedural relations in the form of rules. This formal service specification allows the use of existing description logic reasoners, such as Pellet [35], for the execution of data transformations, inferring new knowledge on the specified ontology at runtime.

The following example SWRL rule states that if a new *heart rate* measurement exceeds the value of “150 bpm”, the patient “Anna” is considered to have an *elevated heart rate*.



Heart_Rate (?heartrate), Patient (Anna),hasMeasurement (Anna,?heartrate),greaterThan (?heartrate,150),-> hasTachycardia (Anna,?heartrate)


Subsequently, services defined in such a manner through the use of SWRL for the definition of preconditions and effects can also be seen as rules. If the service precondition is met, the service executes, resulting in the service effect, which changes the current state of the system. The translation of services to rules provides for the automatic data-driven execution of services based on new events flowing into the system. This is further elaborated by the **inference engine** in Section 4.2 together with a description on the planning algorithms of the **workflow reasoner**, automatically combining service functionality in composite workflows.

An example semantic description of the notification service consists of a precondition stating the registration of a new measurement reading of a patient. This triggers the execution of the service evaluating the new measurement. The result of the execution has two outcomes: (i) the reading exceeds the calculated personal threshold for this patient, in which case, the physician should be notified; or (ii) the threshold is not exceeded, in which case, no alert should be sent. The following semantically describes the service precondition of having a new measurement through the property hasMedicalState (?patient, ?measurement):

<expr:SWRL-Condition rdf:ID=“NewMeasurementCondition”> <rdfs:label>Patient has new medical measurement</rdfs:label> <expr:expressionLanguage rdf:resource=“&expr;#SWRL”/> <expr:expressionObject> <swrl:AtomList> <rdf:first> <swrl:IndividualPropertyAtom> <swrl:propertyPredicate rdf:resource=“http://localhost/AmI/ontology/Medical.owl#hasMedicalState”/> <swrl:argument1 rdf:resource=“#*patient*”/> <swrl:argument2 rdf:resource=“#*measurement*”/> </swrl:IndividualPropertyAtom> </rdf:first> <rdf:rest rdf:resource=“http://www.w3.org/1999/02/22-rdf-syntax-ns#nil”/> </swrl:AtomList> </expr:expressionObject></expr:SWRL-Condition>


Expressing whether or not to send a notification is included in the *conditional effect* of the service result. A *conditional effect* means that depending on the service output, a different effect is possible. For example, a service calculating the new measurement can have as an effect that the threshold is exceeded if the alert is “true” (in which case, a notification should be sent) or not, if “false”. This enables formulating the actual meaning of the service output alert. Without semantics, the output will be merely “true” or “false”, which does not provide the user or the application with the necessary information to trigger a specific action. The following is an example of a condition on the service effect stating the case where the service output *alert* is “false”:

<process:Result rdf:ID=“ThresholdNotExceeded”> <process:inCondition> <expr:SWRL-Condition rdf:ID=“AlertOff”> <expr:expressionLanguage rdf:resource=“&expr;#SWRL”/> <expr:expressionObject> <swrl:AtomList> <rdf:first> <swrl:BuiltinAtom> <swrl:builtin rdf:resource=“http://www.w3.org/2003/11/swrlb#equal”/> <swrl:arguments> <rdf:List> <rdf:first rdf:resource=“#*alert*”/> <rdf:rest> <rdf:List> <rdf:first rdf:datatype=“http://www.w3.org/2001/XMLSchema#string”>    *false* </rdf:first> <rdf:rest rdf:resource=“http://www.w3.org/1999/02/22-rdf-syntax-ns#nil”/> </rdf:List> </rdf:rest> </rdf:List> </swrl:arguments> </swrl:BuiltinAtom> </rdf:first> <rdf:rest rdf:resource=“http://www.w3.org/1999/02/22-rdf-syntax-ns#nil”/> </swrl:AtomList> </expr:expressionObject> </expr:SWRL-Condition> </process:inCondition> <process:hasEffect> … </process:hasEffect></process:Result>


In case the *conditional result* has “true” for the *alert* output, the service effect describes its actual meaning, *i.e.*, a medical threshold has been exceeded:

<process:Result rdf:ID=“ThresholdExceeded”> <process:inCondition> … <rdf:first rdf:datatype=“http://www.w3.org/2001/XMLSchema#string”>*true*</rdf:first> … </process:inCondition> <process:hasEffect> <expr:SWRL-Expression rdf:ID=”AlertOnEffect”> <expr:expressionLanguage rdf:resource=”&expr;#SWRL”/> <expr:expressionObject> <swrl:AtomList> <rdf:first> <swrl:IndividualPropertyAtom> <swrl:propertyPredicate  rdf:resource=“http://localhost/AmI/ontology/Medical.owl#hasThreshold”/> <swrl:argument1 rdf:resource=”#*measurement*”/> <swrl:argument2 rdf:resource=“#*thresholdMeasurement*”/> </swrl:IndividualPropertyAtom> </rdf:first> <rdf:rest> <swrl:AtomList> <rdf:first> <swrl:BuiltinAtom> <swrl:builtin rdf:resource=“http://www.w3.org/2003/11/swrlb#lessThan”/> <swrl:arguments> <rdf:List> <rdf:first rdf:resource=”#*thresholdMeasurement*”/> <rdf:rest> <rdf:List> <rdf:first rdf:resource=”#*measurement*”/> <rdf:rest rdf:resource=“http://www.w3.org/1999/02/22-rdf-syntax-ns#nil”/> </rdf:List> </rdf:rest> </rdf:List> </swrl:arguments> </swrl:BuiltinAtom> </rdf:first> <rdf:rest rdf:resource=“&rdf;#nil”/> </swrl:AtomList> </rdf:rest> </swrl:AtomList> </expr:expressionObject> </expr:SWRL-Expression> </process:hasEffect></process:Result>


Based on this description, the proposed AmI framework in this paper is able to automatically register the deterioration of the patient's state, analyze the consequences and trigger the notification of the responsible physician, potentially executing additional services if required. This process is described in Section 4.2.

#### 5.1.2. Semantic Description of Device QoS Parameters

Once the pool of required services for notifying the responsible physician has been selected, it is possible that some of them can be executed on multiple devices having diverse QoS parameters. The AmI framework should be able to automatically select the most fitted device, depending on generic requirements, such as minimum execution time, and cost and context-aware criteria, such as the current location of the physician or the screen size of his smart phone. In this respect, following the analogy of semantically describing the service functionally enabling automatic discovery, the semantic enrichment of the device QoS parameters allows for their automatic alignment to the end user's current context and future objectives. Thus, relevant information captured by the AmI framework is pushed proactively and presented in a user-specific and context-aware way, supporting the situational awareness of the actors involved.

**Figure 5.**
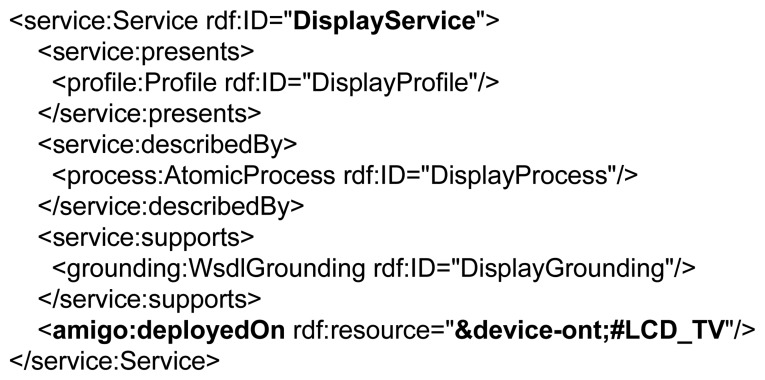
Extension of the standard OWL-Sservice description with the specification of a device on which the service is deployed.

**Figure 6.**
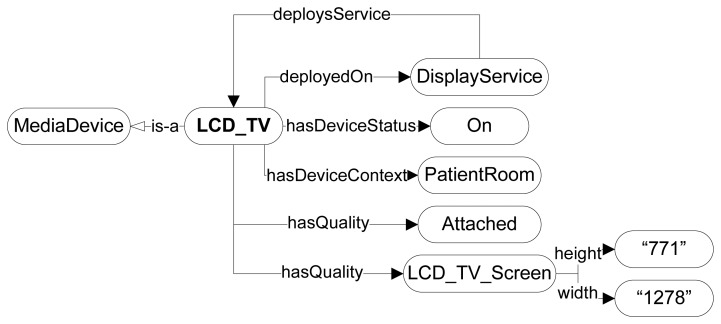
Contextual definition of an LCD TV, which is a media device able to display information in a specific patient room on a 1,278 × 771 screen.

The OWL-S service description is extended with the Amigo [36] device ontology. It defines deployment properties between a device and its running services. Amigo provides support for the description of the device and user context, QoS parameters and communication protocols, such as universal plug and play (UPnP), service location protocol (SLP), Java RMIand simple object access protocol (SOAP). Using the Amigo ontology, we extended the OWL-S service description consisting of the standard OWL-S profile, process and WSDL grounding with the specification of a device presented in Figure 5. In this way, a *display service* additionally defines the actual device instance that it is deployed on (e.g., *LCD TV screen*). This explicit deployment specification enables runtime selection of a service out of several equivalent services, depending on its device QoS parameters. The example in Figure 6 presents the modeling of a *display service* deployed on an *LCD TV device*, which is an instance of a *MediaDevice* concept having properties, such as *location, screen parameters, mobility* features and *device status*. This results in the definition of multiple semantically equivalent *display services* running on different devices. The dynamic selection of a specific device is performed by the **service mapper** detailed in Section 4.3 and will depend on the match between the semantically defined device QoS parameters and the physician's location and preferences.

### 5.2. Medical Patient State Ontology

Additionally to the formal service and device descriptions, this paper also proposes the definition of a semantic patient profile, AmI ontology, in order to guarantee personalized monitoring based on medical profile and real-time measurements. Section 5.2.1. provides a description of a medical patient profile, using an ontology, able to classify patients in different CHF disease risk stages. The method is evaluated in Section 5.2.2. on an actual patient case.

#### 5.2.1. Description of the CHF Classification

**Figure 7.**
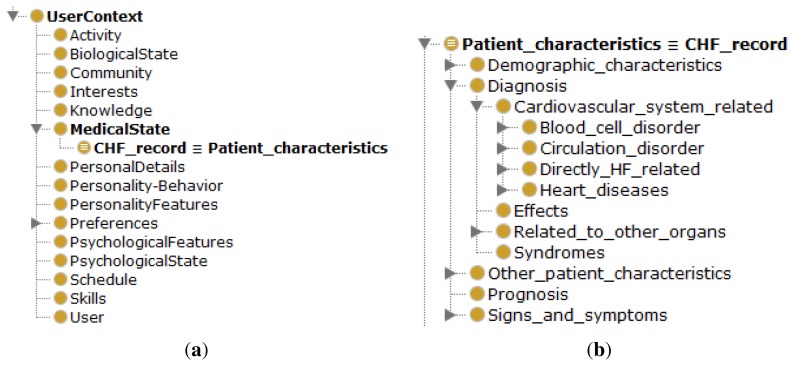
Ontological definition of a patient's medical state according to a specific model for the heart failure disease. (**a**) User Context; (**b**) Patient Record for congestive heart failure.

The Amigo ontology not only supports the definition of a device context, but also of a user context. Presented in Figure 7a, it includes basic profile information, such as the user's activity, schedule and personal details. However, this profile lacks information on his medical state, such as is present in an EHR and, more specifically, a CHF-related description required by the proposed AmI framework. The existing *HF ontology* [37,38] presents a taxonomic overview of the heart failure domain consisting of 200 classes describing HF related concepts. Apart from basic HF concepts, it includes properties that characterize patients, all relevant diagnostic examinations and tests, as well as treatment procedures. Additional concepts are related to the cardiovascular system and other organs connected with HF. Examples are “Cardiac_hypertrophy”, “Blood_pressure_signs”, “Heart murmurs”. Although these concepts are sufficient for describing the CHF-related profile parameters the required automatic classification of patients in risk stages according to the Framingham Heart Study as outlined in Section 3 is still missing. Therefore this ontology is extended with additional concepts and SWRL rules, discussed hereafter, supporting this classification.

**Figure 8.**
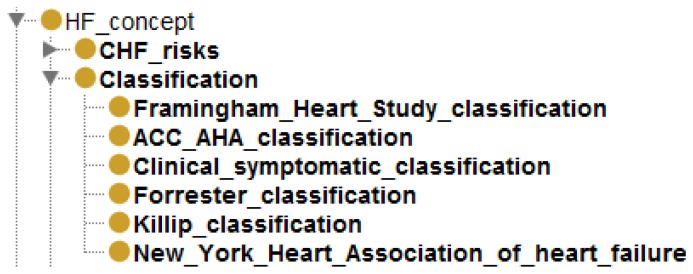
Framingham Heart Study (FHS) added to the HF classification ontology.

In order to model the chronic heart condition of a patient this paper proposes an AmI ontology consisting of an extension of the Amigo ontology with a “MedicalState” concept including a “CHF_record” concept. This concept is defined as an *equivalent ontological concept* to the “Patient_characteristic” concept of the *HF ontology* in Figure 7b. The five basic classes of the *HF ontology*, including the provided extension by the AmI framework are:

- **HF concept:** The key taxonomy consisting of the HF terminology, including the risks for CHF, medical synonyms and types of classification (ACC/AHA, clinical symptomatic, Forrester, Killip, New York Heart Association of heart failure symptoms). We extended this classification taxonomy with an additional class, the “Framingham_Heart_Study _classification”, in order to support the previously mentioned (Section 3) Framingham Heart Study (FHS) risk factors visualized in Figure 8. The risk factors are translated into SWRL rules, automatically classifying patients in specific CHF risk stages based on the four-year probability of congestive heart failure. This probability is calculated by the **monitoring application**, where each medical parameter gets assigned a number of points (FHS points) based on its value (a detailed example is in Section 5.2.2).
- **Patient_characteristic:** Clinical data in the patient's HF medical record, such as demographic characteristics, possible diagnoses, possible signs and symptoms, prognosis and other characteristics. The **Monitoring Application** assembles this data for the evaluation of the specific “Framingham_Heart_Study_classification”. As mentioned before, this concept is linked to the Amigo ontology through sub-classing of a user's medical state.
- **Testing:** Knowledge regarding physical examinations and tests performed in medical institutions. Each test relevant to HF has properties that denote the measurements for that test and also which disorders it can detect.
- **Treatment:** Medical procedures used in the healing process, including medications, devices, invasive and non-invasive procedures and recommendations regarding HF.
- **Patient:** Factual knowledge about particular patients.

#### 5.2.2. Example of Automatic CHF Classification

The proposed AmI ontology models the patient's medical state focusing on the CHF scenario. The additionally defined SWRL rules monitor his current condition, suggesting a possible classification according to the Framingham Heart Study (FHS) risk stage. In case of deteriorated vital signs, notification alerts are sent to the responsible physician. Additionally, it would be possible to extend the CHF ontology with proposed treatments and required tests, which will expand the scope of the workflows. However, these should be verified beforehand by medical personnel.

An example patient profile is a 67-year-old man with documented coronary heart disease struggling with an elevated heart rate and high blood pressure. The AmI ontology enables the registration of his current heart rate and blood pressure measurements and an overview of passed difficulties, calculating a personalized FHS risk stage classification. This is provided by the risk assessment algorithms of the **monitoring application** [5] and the SWRL rules evaluating his FHS risk stage without having to specify actual values and, thus, deviating from the default thresholds in Table 1.

The following is an example calculation of this patient's FHS risk stage as supported by the AmI ontology, calculated by the **monitoring application** and automatically classified by the defined SWRL rules. In order not to make things too complicated, the model does not include vital capacity and chest X-ray results. The FHS points for each medical parameter can be found in Table 2 and the FHS risk stage classification based on the four-year probability of CHF in Table 3.

- The patient is 67 years of *age*. Using the following rule, he will be classified in the “young elderly” *age group* between 65–69, resulting in four FHSs:

Age_group (Young_elderly_(65--69)),StartAge (Young_elderly_(65--69),65), EndAge (Young_elderly_(65--69),69)

Patients (?patient),Age (?patient,?age), Age_group (?age_group),StartAge (?age_group,?start), EndAge (?age_group,?end),lessThanOrEqual (?start,?age),lessThanOrEqual (?age,?end)-> isAgeGroup (?patient,?age_group)
- He has a *systolic blood pressure* of 173 mm Hg, which puts him in the range between 170 and 189 mm Hg, resulting in three FHS:

Patients (?patient),hasDiagnosis (?patient,systolic_blood_pressure)SystolicBloodPressureValue (systolic_blood_pressure,?sbp_value),lessThanOrEqual (170,?sbp_value),lessThanOrEqual (?sbp_value,189)
- He has a *Heart rate* of 57 bpm which puts him in the range between 55–64 bpm resulting in 1 FHS:

Patients (?patient),hasDiagnosis (?patient,?heart_rate),HeartrateValue (?heart_rate,?hr_value),lessThanOrEqual (55,?hr_value),lessThanOrEqual (?hr_value,64)
- He does not have *systolic hypertension* (hasDiagnosis (?patient, Systolic_hypertension)) or *valvular disease* (hasDiagnoasis (?patient, Valvular_heart_disease)), but suffers from*coronary disease*, resulting in eight FHS:

Patients (?patient),hasDiagnosis (?patient,Coronary_heart_disease)
- His ECG shows a *left ventricular hypertrophy* resulting in four FHS:

Patients (?patient),hasDiagnosis (?patient,Left_ventricular_hypertrophy)
- Finally, there are no signs of *diabetes*, which stays at zero FHS.

The end result calculated by the **monitoring application** using these measurements is 20 FHS points, which will result in the evaluation of the following rule classifying the patient with at least a 16% probability of suffering from CHF within four years:

Patients (?patient),CongestiveHeartFailurePoints (?patient,?points)lessThanOrEqual (?points,20), lessThan (18,?points),-> SuggestedDiagnosis (?patient,Heart_failure),CHF (4-Year)Probability (?patient,16)


The *CHF (4-Year)Probability* property is used to automatically classify patients in one of the *Framingham_Heart_Study_classifications* stages (“Framingham_16”) using the equivalent class property:

Patients (?patient),CHF (4-Year)Probability (?patient,16),SuggestedDiagnosis (?patient,Heart_failure),-> HFClassification (?patient,Framingham_16),hasDiagnosis (Framingham_16,Heart_failure)


Another way of calculating the predicted probability of heart failure is by using the coefficients in Table 4 from the model as follows:

(1) $$\begin{array}{l} \begin{array}{l} p\left(\text{xbeta}\right)=1/\left(1+\text{exp}\left(-\text{xbeta}\right)\right),\\ \text{xbeta}=\text{Intercept}+\text{Sum}\left(\text{RegressionCoefficient}\times \text{RiskFactor}\right) \end{array}\\ \begin{array}{l} \text{xbeta}=-9.2087+\left(0.0412\times \text{Age}\right)+\left(0.9026\times \text{LVH}\right)\\ +\left(0.0166\times HR\right)+\left(0.00804\times \text{SystolicBP}\right)\\ +\left(1.6079\times \text{MyocardialInfarction}\right)+\left(0.9714\times \text{HeartMurmur}\right)\\ +\left(0.2244\times \text{Diabetes}\right) \end{array}\\ \begin{array}{l} \text{xbeta}=-9.2087+\left(0.0412\times 67\right)+\left(0.9026\times 1\right)+\left(0.0166\times 57\right)\\ +\left(0.00804\times 173\right)+\left(1.6079\times 1\right)+\left(0.9714\times 0\right)+\left(0.2244\times 0\right)\\ =-9.2087+2.7604+0.9026+0.9462+1.39092+1.6079\\ =-1.60068 \end{array}\\ p\left(-1.60068\right)=1/\left(1+\text{exp}\left(1.60068\right)\right)=1/5.9564=0.1679 \end{array}$$

This indicates a four-year probability of heart failure of 17%, an estimate quite close to the obtained 16% using the point score system (although it is located between 20 (16%) and 22 FHS points (22%)).

Whenever the automatic classification is effective, any service having a HFClassification (?patient, Framingham_Heart_Study_classification) as a precondition (Section 4.1) will be triggered for execution. Additionally, it is also possible to query the ontology specifically for patients belonging to the “Framingham_16” risk stage or, perhaps more interesting, the ones exceeding it, having a diagnosis of “Heart_failure”.



Patients (?patient),HFClassification (?patient,Framingham_16),hasDiagnosis (Framingham_16,Heart_failure)


## 6. Framework Evaluation Results

This section refers to the supportive technologies specific to the proposed implementation of the remote monitoring AmI framework and validates it in terms of the introduced scenario in Section 3, followed by an evaluation of its performance and a discussion on some preliminary clinical studies.

### 6.1. Supportive Technologies

As mentioned in the introductory Section 1, several devices and technologies are available, supporting the design of an AmI framework.

- *Medical devices and sensors*: In our reference implementation, the supported measurements are:
  – blood pressure (BP) and heart rate (HR) (A&D UA-767PBT Blood Pressure Monitor acquiring BP (systolic, diastolic and mean arterial) measurements and HR, transmitted via Bluetooth),
  – SpO_2_ (Nonin Avant 4000 Digital Pulse Oximeter providing real-time measurements of HR and SpO_2_, transmitted via Bluetooth)
  – Body weight (A&D UC-321PBT weight scale measuring the person's weight, transmitted via Bluetooth) and
  – 12-lead ECG monitoring (Welch Allyn Cardio Perfect 12 Lead ECG Recorder, transmitting the recorded ECG via a fiber optic cable).
- *ILS*: Commonly used techniques involve RFID tags, triangulation algorithms based on Wi-Fi signal (strength, angle, distance, attenuation), infrared and visible light communication and ultrasound waves [39]. Depending on the cost, the required precision and the use case-specific parameters, various solutions or combinations are applied. Our reference implementation utilizes the commercially available system Ekahau RTLS 4.3 using the “T301-A” WiFi tag for locating persons or assets.
- *Monitoring application*: A full description and evaluation of this application, which enables the recording of the patient bio signals and hosting risk assessment algorithms, is provided in [5]. Section 5.2.2. provides an example of its integration with the AmI ontology evaluating the patients’ CHF risk stage.
- *Ontology-driven application intelligence*: The applied medical and device ontologies define a formal representation of knowledge by a set of key domain concepts and the relationships between those concepts, enabling the reuse of the medical knowledge and device interoperability (described in detail in Section 5 and illustrated in Figure 2). The advantages of the semantic enhancement are:
  (1) *Automatic classificationof patients in specific CHF risk stages*, depending on their personal medical health record and real-time monitoring data.
  (2) *Data-driven notification* of the responsible physician through the the triggering of generic rules on the personalized patient CHF risk stage.
  (3) *Automatic selection of services* into composite workflows depending on the semantic description of their functionality, providing for the necessary information required for notifying the medical personnel of the patients’ state.
  (4) *Context-aware dynamic selection of devices* for executing the required services based on the semantic description of their QoS parameters matched to the current context of the responsible physician that needs to be notified.

### 6.2. Validation of the Smart and Adaptable Notification Scenario

In order to evaluate the proposed dedicated AmI framework, a patient monitoring and clinician notification scenario is implemented. The following (medical) services are enriched with semantic annotations using the latest version of OWL-S 1.2 as described in Section 5.1.1.

- **Medical measurement**services: register various medical readings of the patients (e.g., pulse rate, blood pressure, ECG). They can be used either with a pull model (polling it to get a measurement) or a push model (generating an event each time there is a new measurement).
- **Notification** services: generate alert notifications when a measurement gets outside of specified CHF risk stage thresholds. These thresholds are evaluated through reasoning on the developed CHF ontology and rules and can be set and modified dynamically based on the personal record of the patient.
- **User location** services: localize a specific user using the *ILS* system.
- **Display** services: display patient measurements on a screen. This service is supported by several devices, such as the physician's personal tablet and television screens in different rooms.
- **Redirection** services: localize the nearby display device.
- **Summarize measurement** services: extract only vital or high level information out of complex medical data in order to be able to present it on a small screen device.
- **Patient record** services: provide patient information, including the treating physician.
- **Generate display** services: generate actual information to be displayed using **summarize measurement** services if the device has a small screen size.

As mentioned in the previous section, the communication between the architectural components of the AmI framework is supported through the eventing mechanism of the **execution environment.** The following scheme provides a brief description of the internal functionality of the AmI during the realization of the physician notification scenario in Figure 1 using the available semantically-enhanced services. It is divided in an *initialization stage*, which is triggered only once during the start-up of the the AmI framework, and a *runtime phase* that executes each time new data is captured by the framework.

(1) Initialization phase:
  (a) **Service manager:**classifies the semantic descriptions of the available services running on the different devices into groups of semantically equivalent services. For example, several “display” services running on devices with different characteristics are grouped into a common “display” service description.
  (b) **Inference Engine:**
    - translates the common service descriptions into SWRL rules that are loaded into the knowledge base of the Pellet reasoner,
    - loads additional patient data and rules defined by the developed ontologies into the knowledge base of the Pellet reasoner.
(2) Runtime phase (describes the scenario steps from Section 3):
  (a) **Inference engine:** processes real-time patient data captured by the “medical measurement” services. The defined service and *user rules* are automatically evaluated to detect possible deviations from normal values or classification in CHF risk stages. In the event of an exceeded threshold (Step 1) met by the precondition of the “notification” service, the **inference engine** triggers its execution by registering this in the **execution environment.**
  (b) **Execution environment:** sends the triggered service event to the **workflow reasoner**.
  (c) **Workflow reasoner:** evaluates the necessary data and conditions in order to execute the “notification” service. During the workflow planning, it identifies the required information on the specific patient's (“patient record”), actual service able to display the notification (“display”), the definition of the information to display (‘’generate display”) and localization of the responsible physician to whom to send the notification (“user location”). These services are linked together using semantic matching techniques.
  (d) **Service mapper:** constructs a composite service for execution by selecting the corresponding devices linked to the pool of required service interfaces.
    - For each semantic service description, an actual service is selected based on its device QoS characteristics. These are prioritized using weights based on device status (on/off), location (relative to the physician) and screen size (the bigger the better). As several “display” devices are available with different screen sizes and locations, it detects the requirement for determining the actual location (“user location”).
      - **Execution engine:** executes the “user location” service, localizing the responsible physician (Step 2).
      - **Execution environment:**
        – loads the results of this service in the knowledge base of the **inference engine**,
        – notifies the **service mapper** of the new results.
    - Selects the physician's tablet as the nearest device for executing the “display” service.
    - As its screen size is bellow a defined threshold (SWRL *service rule*), additional conditions are detected (preconditions of the “display” service) to provide a summarized version of the medical information (“summarize measurement”) and to redirect the physician to a device with better screen capabilities (“redirect”).
      - **Execution environment:** triggers the missing information event sent to the **workflow reasoner**.
      - **Workflow Reasoner:** selects the required “Summarize Measurement” and “Redirect” services.
  (e) **Service mapper:** disposes of all the services in order to construct a composite service for execution.
  (f) **Execution engine**:
    - handles the execution of the workflow by alerting the responsible physician (“summarize measurement”, “generate display”, “display”) (Step 3),
    - identifies any surrounding devices capable of remotely displaying vital signs (“redirect”) and depending on their characteristics a summary or complete overview of the patient's state is provided; the clinician is redirected to a larger display (Step 4) in order to analyze the vital signs in detail (Step 5).

### 6.3. Performance and Scalability Evaluation

In order to validate the AmI in terms of performance and scalability, a composite service workflow is designed, consisting of six levels, a breadth of three services and 10 different service nodes multiplied by 50 semantically equivalent services per node. Different measurements are observed: the initial classification of service instances into groups of equivalent semantic descriptions, the selection of a specific service functionality by the **service manager**, the construction of a semantic mashup by the **workflow reasoner**, the construction of an executable process by the **service mapper** and the runtime recovery during failure by the **execution engine**. The reaction time of the **inference engine** is not taken into consideration, as the supporting Pellet reasoner reacts instantly the moment a rule is triggered. The measurements are performed on two types of machines: a PC with 2.40 GHz Intel Core 2 Duo and 4 GB RAM and the iLab.t VirtualWall [40]. This is a large-scale generic test environment for advanced networks, distributed software and service emulation and evaluation, supporting scalability research. The VirtualWall facilities consist of 100 nodes (dual processor, dual core servers, 6 × 1 Gb/s interfaces per node) interconnected via a non-blocking 1.5 Tb/s VLAN Ethernet switch and a display wall (20 monitors) for experiment visualization. Each server is connected with four or 6 GB Ethernet links to the switch.

The PC experiments consist of a pool of 50 services (five equivalent services per node), while the VirtualWall, having more processing power, is scaled up to 500 services (50 equivalents per node). The following results reflect the optimization of the performance of the different components thanks to the automatic grouping of equivalent service descriptions:

(1) **Service Manager, loading and grouping of semantic service descriptions:**

**PC****VirtualWall**
No grouping1.7 s1 minGrouping3.5 s5 min
  Although the difference between service loading with or without grouping grows steadily with growing number of services, it is a necessary one-time comparison that considerably speeds up the service matching (**workflow reasoner**), selection (**service mapper**) and recovery from failure (**execution engine**) process, as can be seen in the next paragraphs.
(2) **Service manager, querying for a specific semantic service functionality:**

**PC****VirtualWall**
No grouping2.6 s1 minGrouping0.4 s1s
  Logically, with grouped services, one only needs to consider the 10 service groups, while without grouping, all services need to be queried, resulting in an exponential increase of service querying.
(3) **Workflow reasoner, automatic composition of services into a workflow:**

**PC****VirtualWall**
No grouping287 soverloadGrouping10 s1 min
  The composition time is an accumulation of querying for required service functionality, the actual construction of the service workflow through linking of the matching services to each other and removing of partially constructed alternative paths. With grouping only the groups of equivalent services are considered, while without grouping, all available services are queried resulting in an exponentially growing composition time.
(4) **Service mapper, selection of an actual executable service based on QoS for every service from the constructed workflow:** Independent of the machine it is running on, the selection of a specific service from a group of equivalent services takes between two to 5 ms. The construction of an executable workflow takes 2.5 s in the case of no equivalents and grows to 8 s for 500 services. This increase is due to the growing service pool, as the mashup graph is always the same.
(5) **Execution engine, workflow adaptation due to service failure:** In case of a failed service, there are two possible scenarios depending on whether there is an equivalent service for the failed one or not. In the first case, the **service mapper** selects an equivalent service (different QoS), and the execution continues. This takes half a second on average. If there is no equivalent service, the **workflow reasoner** needs to reconstruct the failed part of the workflow, resulting in an adapted workflow. In the worst case, when all the equivalent services are broken and time is lost to try them all before the **workflow reasoner** is triggered, this could take up to 5 s. This requires alternative strategies optimizing the workflow reconstruction.

The results show that the performance and scalability of the designed planning algorithms are strongly dependent on the grouping of the semantically equivalent services. As the **workflow reasoner** only considers the groups of services, there is no time loss during workflow planning with the growing number of equivalent services. This allows the **service mapper** to select a specific service for execution, depending on its device QoS and the context of the patient and his physician.

### 6.4. Clinical Studies

For the evaluation and validation of our platform, we have carefully designed and executed a range of studies, both qualitative and quantitative. These included the evaluation of results by real end-users in a controlled environment (lab) conducted by the design of and execution of test cases with known results. More specifically, 10 randomized trials were setup, simulating the attenuated ECG voltage, resulting in a decreased amplitude of QRScomplexes, P-waves and a shortened duration of QRS complexes and QTintervals, with significant diagnostic implications, low oxygen saturation and out ranged values exceeding the thresholds indicated in Table 1. BMI and age were provided by the EHR system.

In parallel, several qualitative methods were also employed, which included both interviews (two medical doctors from the Emergency Department at the University Hospital of Heraklion) and a focus group that includes two male CHF patients (aged 65 and 69) diagnosed with no other major chronic diseases, being remotely monitored by a mixed group of experts, including one internist and one cardiologist/intensivist from the University Hospital. The length of the follow-up study is set to two months. However, there are no symptoms requiring any intervention, yet. The interviews identified the need for such a system, assessing the impact on the following key elements: (i) a reduced time to physician awareness; (ii) an increased quality of care; (iii) an improved quality of life/patient satisfaction; (iv) early discharge from the ICU; (v) limited bed days of care; and (vi) reduced emergency department visits.

## 7. Discussion

Context-aware medical remote monitoring and decision support systems are of critical importance. A 24/7 patient monitoring makes medical departments, such as the ICU, very data-intensive. Physicians are expected to sift through the huge amount of documented data and respond in a timely manner to deteriorations in the patient's state. The proposed AmI framework is the first step towards a remote monitoring decision support system that takes over and speeds up the continual processing of tons of data, assisting physicians during patients’ treatment. Context-aware notifications are optimized and tailored to the physician's location and device characteristics. It is enhanced with the adoption of SOAs, which offer the advantage of building component-based systems reusing medical services. In a dynamic environment, where one cannot always predict the desired functionality, all kinds of custom-made compositions can be built from scratch, meeting the users’ needs without the continuous interaction between medical staff and developers. This computer-aided approach is accomplished through the adoption of OWL-S for the enrichment of the medical services with semantics. Based on their semantic description, the **workflow reasoner** of the AmI is able to determine which services can be joined together to execute a *composite task* in the case of required service inputs. This process is triggered by rules defining thresholds on patients’ medical parameters monitored by the **inference engine**. The components constantly capture new information on the patient's state, resulting in automatic real-time classification in *CHF risk stages*. The classification triggers the notification of the responsible physician. Once an event is triggered, a *workflow of medical services is automatically constructed* by the **workflow reasoner**, gathering additionally required information. As the semantic services are classified into groups of equivalent services running on different devices, the **service mapper** is responsible for selecting a specific device for execution. This *context-aware* component provides strategies for *QoS comparison* of the *device characteristics* defined through an ontology. Depending on the responsible physician's location, it sends critical notifications to his tablet or other available devices in his proximity, offering a better screen size for viewing the vital signs of the patient. This information is redirected to nearby devices whenever the physician changes location, resulting in a *dynamic remote monitoring* system. Additionally, in order to guarantee the successful execution of the composite medical service, the *recovery procedure* of the **execution engine** guards service failures, selecting/constructing an alternative/composite service, finalizing the execution.

The use of ontologies not only supports the *semantic description* of *services and device QoS parameters*, but also the definition of *patient and physician profiles*. Extending existing medical profiles with classification schemes for specific diseases, such as congestive heart failure, enables the automatic generation of rules monitoring the patient's state and triggering events when thresholds are exceeded. Those thresholds are tailored to the specific patient's profile, resulting in *personalized CHF risk stage classification* and a *notification* system supported by the AmI's **inference engine.**

Current evidence supports the feasibility of such an approach, while our solution is expected to relax the determinants of early discharge from the intensive care unit after cardiac operations with no risk of possible complications (since patients are still remotely monitored) compared with patients treated with a standard strategy. In addition, home monitoring is also expected to reduce the bed days of hospitalized care (the patient can be efficiently monitored in his own place of care) and the number of emergency department visits. Apart from the economic impact for CHF management, patient satisfaction and quality of life are of utmost importance.

Future research should include more studies, including more participants coming from diverse patient populations and a longer study period in order to accurately measure the long-term effectiveness of the proposed system.

## 8. Conclusions

This paper presents the development of an ambient intelligence (AmI) framework supporting real-time monitoring of patients diagnosed with congestive heart failure (CHF). A medical patient's profile together with the CHF specification are formally described through an ontology. These semantic profiles enable the automatic classification of patients into specific CHF risk stages depending on their medical history and current medical measurements, personalizing the provided healthcare. Services monitoring vital patient signs register the critical deterioration of the patient's state, resulting in the execution of alerting services. In order to provide the responsible physician with timely and complete information on the specific patient, planning algorithms automatically assemble medical workflows out of existing services, collecting the required patient data. These (medical) services are semantically enriched, enabling the automatic selection of the most suitable device for execution, depending on its availability, location, screen parameters and other QoS. Dynamic notification mechanisms take into account the physician's location and succeed in remotely displaying the full vital sign monitoring application on the device of his/her choice, ensuring in-time intervention in case of an emergency. The medical workflows are dynamically adapted in order to respond to a changing context, such as the patient state, physician location, new services/devices, additional disease specification rules and failure/overload of the network infrastructure. In order to illustrate the AmI framework, a clinical scenario related to CHF is validated, in which a deterioration of the patient's state results in the localization and notification of medical personnel presenting *ad hoc* information on the patient's condition on the best suited device within the physician's reach.

## Figures and Tables

**Table 1.** Alert detection parameters and corresponding default thresholds. HR, heart rate; BP, blood pressure.

**Measurement**	**Device**	**Detection Threshold**
low SpO_2_	Pulse Oximeter	SpO_2_ < 90%
bradycardia	Pulse Oximeter	HR < 40 bpm
tachycardia	Pulse Oximeter	HR > 150 bpm
HR change	Pulse Oximeter	| Δ HR/5 min| > 19%
HR stability	Pulse Oximeter	max HR var past four readings >10%
BP change	BP Monitor	systolic or diastolic change >±11%

**Table 2.** Predictors for congestive heart failure within four years for men aged 45 to 94 years (with coronary disease, hypertension or valvular disease; the model is without vital capacity and chest X-ray results).

**FHS Points**					

	**0**	**1**	**2**	**3**	**4**
Age, y	45–49	50–54	55–59	60–64	65–69
Systolic BP, mm Hg	<120	120–139	140–169	170–189	190–219
HR, bpm	<55	55–64	65–79	80–89	90–104
LVH on ECG	No				Yes
Coronary heart disease	No				
Valve disease	No				
Diabetes	No	Yes			

**FHS Points**					

	**5**	**6**	**7**	**8**	**9**

Age, y	70–74	75–79	80–84	85–89	90–94
Systolic BP, mm Hg	>219				
HR, bpm	>104				
LVH on ECG					
Coronary heart disease				Yes	
Valve disease	Yes				
Diabetes					

**Table 3.** Probability of congestive heart failure within four years for men aged 45 to 94 years)

**FHS Points**	**4-Year Probability, %**	**FHS Points**	**4-Year Probability, %**
5	1	24	30
10	3	25	34
12	3	26	39
14	5	27	44
16	8	28	49
18	11	29	54
20	16	30	59
22	22		

**Table 4.** Pooled logistic regression model for men with coefficients (the model is without vital capacity and chest X-ray information). OR, odds ratio; CI, confidence interval; LVH, left ventricular hypertrophy; CHD, congenital heart disease.

**Variables**	**Units**	**Regression Coefficient**	**OR (95% CI)**	**P**
Intercept		−9.2087		
Age	10 y	0.0412	1.51 (1.31–1.74)	<0.001
LVH	Yes/no	0.9026	2.47 (1.31–3.77)	<0.001
HR	10 bpm	0.0166	1.18 (1.08–1.29)	<0.001
Systolic BP	20 mm Hg	0.00804	1.17 (1.04–1.32)	0.007
CHD	yes/no	1.6079	4.99 (3.80–6.55)	<0.001
Valve disease	Yes/no	0.9714	2.64 (1.89–3.69)	<0.001
Diabetes	Yes/no	0.2244	1.25 (0.89–1.76)	0.2

## References

[1] Gao T., Greenspan D., Welsh M., Juang R., Alm A. Vital Signs Monitoring and Patient Tracking over a Wireless Network.

[2] Kostomanolakis S., Kavlentakis G., Sakkalis V., Chronaki C., Tsiknakis M., Orphanoudakis S. Seamless Integration of Healthcare Processes Related to Image Management and Communication in Primary Healthcare Centers.

[3] White L., Terner M. (2001). E-health, phase two: The imperative to integrate process automation with communication automation for large clinical reference laboratories. J. Healthc. Inf. Manag..

[4] Lloyd-Jones D., Larson M., Leip E., Beiser A., D’Agostino R., Kannel W., Murabito J., Vasan R., Benjamin E., Levy D. (2002). Lifetime risk for developing congestive heart failure. Circulation.

[5] Kartakis S., Tourlakis P., Sakkalis V., Zacharioudakis G., Stephanidis C. Enhancing the Patient Experience through Ambient Intelligence Applications in Health Vare.

[6] Greenes R. (2007). Clinical Decision Support: The Road Ahead.

[7] Mamlin B., Overhage J., Tierney W., Dexter P., McDonald C. (2007). Clinical Decision Support within the Regenstrief Medical Record System. Clinical Decision Support Systems: Theory and Practice.

[8] Teich J., Glaser J., Beckley R., Aranow M., Bates D., Kuperman G., Ward M., Spurr C. (1999). The Brigham integrated computing system (BICS): Advanced clinical systems in an academic hospital environment. Int. J. Med. Inform..

[9] Van Den Bossche B., van Hoecke S., Danneels C., Decruyenaere J., Dhoedt B., de Turck F. (2008). Design of a JAIN SLEE/ESB-based platform for routing medical data in the ICU. Comput. Methods Programs Biomed..

[10] Hristoskova A., Moeyersoon D., van Hoecke S., Verstichel S., Decruyenaere J., de Turck F. (2010). Dynamic composition of medical support services in the ICU: Platform and algorithm design details. Comput. Methods Programs Biomed..

[11] Lo C.C., Chen C.H., Cheng D.Y., Kung H.Y. (2011). Ubiquitous healthcare service system with context-awareness capability: Design and implementation. Expert Syst. Appl..

[12] Pung H.K., Gu T., Xue W., Palmes P.P., Zhu J., Ng W.L., Tang C.W., Chung N.H. (2009). Context-aware middleware for pervasive elderly home care. IEEE J. Sel. Areas Commun..

[13] García-Sánchez P., González J., Mora A.M., Prieto A. (2012). Deploying intelligent e-health services in a mobile gateway. Expert Syst. Appl..

[14] Philips Healthcare Neonatal Event Review. http://www.healthcare.philips.com/main/products/patient_monitoring/pro-ducts/neonatal_event_review/.

[15] Philips Healthcare Protocol Watch Project. http://www.healthcare.philips.com/main/products/patient_monitoring/pro-ducts/protocol_watch/.

[16] Philips Healthcare On-Line Electronic Help. http://www.healthcare.philips.com/main/products/patient_monitoring/pro-ducts/oleh/.

[17] Philips Healthcare IntelliVue Guardian Early Warning Score. http://www.healthcare.philips.com/main/products/patient_monitoring/pro-ducts/intellivue_guardian_ews/.

[18] Philips Healthcare Event Surveillance. http://www.healthcare.philips.com/main/products/patient_monitoring/pro-ducts/event_surveillance/.

[19] Thestrup J., Gergely T., Beck P. Exploring New Care Models in Diabetes Management and Therapy with a Wireless Mobile eHealth Platform.

[20] Rocha A., Martins A., Freire Junior J., Kamel Boulos M., Vicente M., Feld R., van de Ven P., Nelson J., Bourke A., óLaighin G. (2011). Innovations in health care services: The CAALYX system. Int. J. Med. Inform..

[21] Maniatopoulos G., McLoughlin I., Wilson R., Martin M. (2009). Developing virtual healthcare systems in complex multi-agency service settings: The OLDES Project. Electron. J. e-Gov..

[22] Eikerling H., Gräfe G., Röhr F., Schneider W. Ambient Heahltcare System: Using the Hydra Embedded Middleware for Implementing an Ambient Disease Management System.

[23] Eisenhauer M., Rosengren P., Antolin P. A Development Platform for Integrating Wireless Devices and Sensors into Ambient Intelligence Systems.

[24] Amoretti M., Copelli S., Wientapper F., Furfari F., Lenzi S., Chessa S. (2013). Sensor data fusion for activity monitoring in the PERSONA ambient assisted living project. J. Ambient Intell. Humaniz. Comput..

[25] Ram R., Furfari F., Girolami M., Ibañez Sánchez G., Lázaro-Ramos J.P., Mayer C., Prazak-Aram B., Zentek T. (2013). universAAL: Provisioning Platform for AAL Services. Ambient Intelligence-Software and Applications.

[26] Herfet T., Kirste T., Schnaider M. (2001). EMBASSI multimodal assistance for infotainment and service infrastructures. Comput. Graph..

[27] Heider T., Kirste T. (2002). Supporting goal-based interaction with dynamic intelligent environments. ECAI.

[28] Heider T., Kirste T. (2005). Smart environments and self-organizing appliance ensembles. Mob. Comput. Ambient Intell..

[29] Kannel W., D’Agostino R., Silbershatz H., Belanger A., Wilson P., Levy D. (1999). Profile for estimating risk of heart failure. Arch. Intern. Med..

[30] Cândido G., Barata J., Colombo A., Jammes F. (2009). SOA in reconfigurable supply chains: A research roadmap. Eng. Appl. Artif. Intell..

[31] Hristoskova A., Volckaert B., de Turck F. (2012). The WTE+ Framework: Automated construction and runtime adaptation of service mashups. Autom. Softw. Eng..

[32] Möller T. OWLS API-OSIRIS Next..

[33] OWL-S, Semantic Markup for Web Services. http://www.w3.org/Submission/OWL-S/.

[34] SWRL: A Semantic Web Rule Language Combining OWL and RuleML. http://www.w3.org/Submission/SWRL/.

[35] Pellet: OWL 2 Reasoner for Java. http://clarkparsia.com/pellet/.

[36] Vallée M., Ramparany F., Vercouter L. Dynamic Service Composition in Ambient Intelligence Environments: A Multi-Agent Approach.

[37] Jović A., Gamberger D., Krstacic G. (2011). Heart failure ontology. Bio-Algorithms Med-Syst..

[38] Gamberger D., Prcela M., Jović A., Śmuc T., Parati G., Valentini M., Kawecka-Jaszcz K., Styczkiewicz K., Kononowicz A., Candelieri A. Medical knowledge representation within Heartfaid platform.

[39] Chiou Y., Wang C., Yeh S. (2010). An adaptive location estimator using tracking algorithms for indoor WLANs. Wirel. Netw..

[40] iLab.t Virtual Wall. http://ilabt.ibbt.be/.

